# Advances and perspectives in discovery and functional analysis of small secreted proteins in plants

**DOI:** 10.1038/s41438-021-00570-7

**Published:** 2021-06-01

**Authors:** Xiao-Li Hu, Haiwei Lu, Md Mahmudul Hassan, Jin Zhang, Guoliang Yuan, Paul E. Abraham, Him K. Shrestha, Manuel I. Villalobos Solis, Jin-Gui Chen, Timothy J. Tschaplinski, Mitchel J. Doktycz, Gerald A. Tuskan, Zong-Ming (Max) Cheng, Xiaohan Yang

**Affiliations:** 1 Department of Plant Sciences, University of Tennessee, Knoxville, TN USA; 2 Biosciences Division, Oak Ridge National Laboratory, Oak Ridge, TN USA; 3 State Key Laboratory of Subtropical Silviculture, School of Forestry and Biotechnology, Zhejiang A&F University, Hangzhou, Zhejiang China; 4 The Center for Bioenergy Innovation, Oak Ridge National Laboratory, Oak Ridge, TN USA; 5 Department of Genome Science and Technology, University of Tennessee, Knoxville, TN USA; 6 College of Horticulture, Nanjing Agricultural University, Nanjing, Jiangsu China

**Keywords:** Proteomics, Intracellular signalling peptides and proteins

## Abstract

Small secreted proteins (SSPs) are less than 250 amino acids in length and are actively transported out of cells through conventional protein secretion pathways or unconventional protein secretion pathways. In plants, SSPs have been found to play important roles in various processes, including plant growth and development, plant response to abiotic and biotic stresses, and beneficial plant–microbe interactions. Over the past 10 years, substantial progress has been made in the identification and functional characterization of SSPs in several plant species relevant to agriculture, bioenergy, and horticulture. Yet, there are potentially a lot of SSPs that have not been discovered in plant genomes, which is largely due to limitations of existing computational algorithms. Recent advances in genomics, transcriptomics, and proteomics research, as well as the development of new computational algorithms based on machine learning, provide unprecedented capabilities for genome-wide discovery of novel SSPs in plants. In this review, we summarize known SSPs and their functions in various plant species. Then we provide an update on the computational and experimental approaches that can be used to discover new SSPs. Finally, we discuss strategies for elucidating the biological functions of SSPs in plants.

## Introduction

Plant small secreted proteins (SSPs) are less than 250 amino acids (aa) in length and can be actively transported out of plant cells^1,2^. In plants, SSPs have been shown to play important roles in various biological processes such as growth, development, reproduction, resistance to abiotic and biotic stresses, and beneficial plant–microbe interactions^3–5^. In general, 30,000–40,000 protein-encoding genes have been reported in individual plant genomes^6^. Yet hundreds to thousands of SSPs are potentially overlooked in a single plant genome^7^ for two reasons: (1) the SSP space is occupied by many proteins with a length of less than 100 aa^2,8^ and (2) 50% of the discovered secreted proteins in plants do not have a known signal peptide^9^, both of which create difficulties in SSP annotation using traditional computational approaches^10–12^.

In recent years, the increasing volume of genomics data and the continuously evolving machine learning algorithms have boosted the effectiveness of computationally predicting SSPs. Meanwhile, advances in functional genomics research have accelerated the experimental validation of predicted SSPs and the elucidation of their functional roles. As a result, SSP-focused research has become an emerging area with great potential for growth, as reflected by the rapidly increasing number of publications on SSPs in various organisms, including animals, microbes, and plants. Here with a focus on plant SSPs, we first summarize the current understanding of SSP biosynthesis and secretion. We then discuss the structures and functions of representative SSPs that are well characterized in various plant species, including model species, food crops, bioenergy feedstocks, and horticultural plants. We also highlight computational tools, experimental approaches, and their combinations used to identify novel SSPs. Finally, we discuss the strategies that have been or can be used to explore the functions of SSPs.

## Biosynthesis and secretion of SSPs in plants

### Biosynthesis of SSPs

**Fig. 1:**
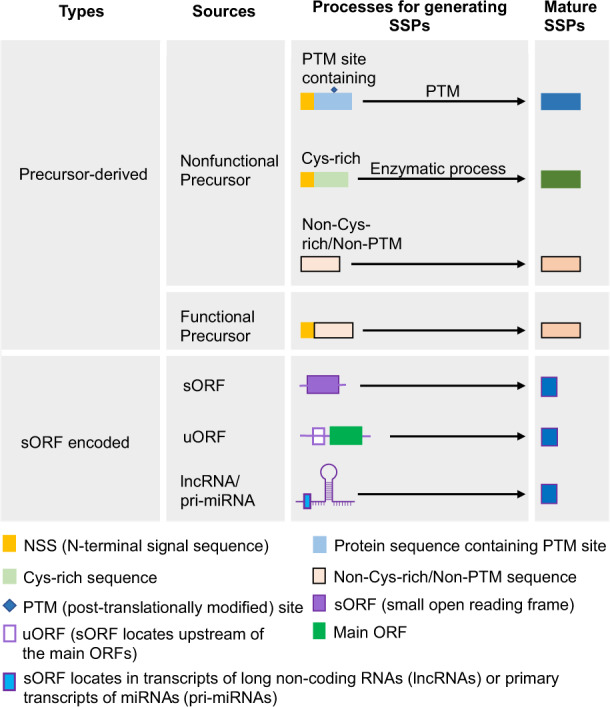
Classification of small secreted proteins (SSPs) in plants. Plant SSPs can be derived from protein precursors, which can be either nonfunctional or functional, or translated from small open reading frames (sORFs). SSPs derived from functional protein precursors often contain an N-terminal signal sequence (NSS), which is removed during maturation. SSPs synthesized from nonfunctional precursors can be further divided into three categories: post-translationally modified (PTM) SSPs, Cys-rich SSPs, and non-Cys-rich/non-PTM SSPs. In addition, SSPs can be encoded by sORFs that locate at upstream of main ORFs (uORFs), in transcripts of long non-coding RNAs (lncRNAs), or primary transcripts of miRNAs (pri-miRNAs). Adapted from ref. ^11^

In plants, SSPs have been found to be produced via multiple alternative pathways, as illustrated in Fig. 1. The majority of the characterized SSPs to date are proteolytic cleavage products synthesized via the removal of an N-terminal signal sequence (NSS; also known as N-terminal signal peptide) and/or a pro-domain from larger protein precursors, which can be either nonfunctional or functional^11,13^. SSPs derived from nonfunctional precursors can be further classified into three subcategories based on features of their mature forms. SSPs belonging to the first subcategory typically consist of less than 20 aa in their mature forms which have few or no cysteine (Cys) residues and contain one to several types of post-translational modifications (PTM), such as tyrosine (Tyr) sulfation, proline (Pro) hydroxylation or Pro glycosylation. Therefore, these SSPs are named PTM SSPs. Several well-studied PTM SSPs in *Arabidopsis thaliana* are involved in plant growth and development, including CLAVATA 3 (CLV3), C-TERMINALLY ENCODED PEPTIDE 1 (CEP1), PLANT PEPTIDE CONTAINING SULFATED TYROSINE 1 (PSY1), and ROOT MERISTEM GROWTH FACTOR 1 (RGF1)^11,14,15^. The second subcategory features SSPs with mature peptides that contain an even number (often ranging from 2 to 16) of Cys residues. These Cys residues are essential for forming the disulfide bonds in the active mature SSPs. Most of the known Cys-rich SSPs are involved in plant–microbe interactions, such as PLANT DEFENSINs (PDFs), nonspecific LIPID TRANSFER PROTEINS (nsLTPs), and KNOTTINs. Meanwhile, several Cys-rich SSPs have been found to regulate plant development, such as S-LOCUS CYSTEINE-RICH PROTEIN/S-LOCUS PROTEIN11 (SCR/SP11) and LUREs^11,15^. The third subcategory contains non-Cys-rich/non-PTM SSPs, which often lack the NSS in their precursor forms and contain Cys, Pro, Tyr, glycine (Gly), lysine (Lys), or other amino acids with dominant roles in conferring the activity of the mature SSPs. SSPs within this subcategory have been primarily found to participate in plant defense responses, with SYSTEMINS (SYS), GRIM REAPER PEPTIDE (GRIp), and PLANT ELICITOR PEPTIDES (PEPs) being the representative examples^11^.

In the past decade, a growing number of plant SSPs has been found derived from functional protein precursors, such as INCEPTINs from *A. thaliana*, *Zea mays*, *Oryza sativa*, and *Vigna unguiculata*, the *Glycine max* SUBTILASE PEPTIDE (Gm-SUBPEP), and the *Solanum lycopersicum* CYSTEINE-RICH SECRETORY PROTEINS, ANTIGEN5, and PATHOGENESIS-RELATED 1 PROTEINS derived peptide 1 (CAPE1)^11^.

In addition to being processed from larger protein precursors, plant SSPs can be directly encoded by small open reading frames (sORFs), which can sometimes locate upstream of the main ORFs (therefore called “uORFs”), within presumed non-coding RNAs (e.g., long non-coding RNAs), or within primary transcripts of miRNAs. These SSPs are denoted as “short peptides encoded by sORFs”, “sPEPs”, or “nonprecursor-derived peptides”^11,16,17^. Some known examples of such SSPs include the uORF2-encoded sucrose control peptide (SC-PEPTIDE) that is required for sufficient sucrose-induced repression of translation in *A. thaliana*^18^, the miPEP171b that regulates root development in *Medicago truncatula*^19^, and ENOD40s that are involved in sucrose use in nitrogen-fixing nodules in *G. max*^20^.

### Mechanisms of SSP secretion

**Fig. 2:**
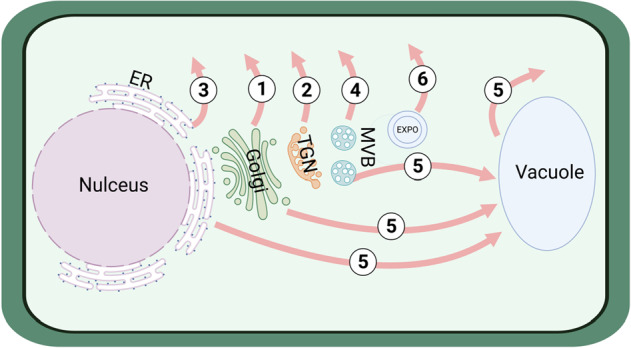
Secretion mechanisms of small secreted proteins (SSPs) in plants. Most N-terminal signal sequence (NSS)-containing SSPs are secreted via the conventional protein secretion (CPS) pathway that begins at the endoplasmic reticulum (ER). SSPs are subsequently routed through either the Golgi apparatus (Golgi) (1) or trans-Golgi network (TGN) (2) before being delivered to the apoplast. Alternatively, some NSS-containing SSPs are secreted by unconventional protein secretion (UPS) routes, including direct transportation from ER to the apoplast (3), transportation through secretory multivesicular bodies (MVBs) (4), and vacuoles (5). Cytosolic leaderless proteins (LSPs) are secreted through the excyst-positive organelle (EXPO) (6). Adapted from ref. ^22^

Our knowledge of plant SSP secretion largely overlaps with our understanding of protein trafficking and secretion, which follows several different mechanisms^21–23^. The majority of plant SSPs with an NSS are secreted via the conventional protein secretion (CPS) pathway (Fig. 2), which is conserved among eukaryotes. Guided by the NSS, SSPs are first transported to the endoplasmic reticulum (ER) where the NSS is removed. These SSPs are then exported to the *cis* side of the Golgi apparatus (Golgi) and further sorted through the Golgi or the trans-Golgi network (TGN). Modifications, such as glycosylation that are required for SSP maturation, occur when SSPs travel through the Golgi. Finally, the mature SSPs are delivered to the apoplast via secretory vesicles or granules^17,22–24^.

However, some NSS-containing SSPs bypass the CPS pathway. They follow unconventional protein secretion (UPS) routes (Fig. 2)^22,23^ while traveling to the extracellular space, usually upon pathogen attack or the exposure to other biotic or abiotic stress conditions^9,24^. The simplest UPS route directly transports these proteins from the ER to the plasma membrane (PM). Alternative UPS routes utilize vesicular carriers, including the secretory multivesicular body (MVB) and vacuole, that can fuse with the PM to release their contents into the apoplast/extracellular space^22^.

In addition, secreted proteins without an NSS (also known as cytosolic leaderless proteins, LSPs), which represent a large proportion of the plant secretome^21^, cannot be processed by the CPS. These proteins have been proposed to be secreted through the excyst-positive organelle (EXPO)—a double-membrane organelle whose formation is Golgi- and TGN-independent. The EXPOs can fuse with the PM to secrete LSPs (Fig. 2)^9,21^.

## Known SSPs and their biological roles in plants

### Known SSPs

Because the genome of model herbaceous plant *A. thaliana* is considered to be better annotated and characterized than other plant species, we focus on known SSP families found in *A. thaliana*. Also, we discuss SSPs that have been identified from several important plant species, including *Z. mays*, *O. sativa*, *S. lycopersicum*, *M. truncatula*, and *Populus trichocarpa*. A large number of SSPs have been computationally predicted in plants, as demonstrated in public databases, including OrysPSSP^5^, PlantSSP^25^, and MtSSPdb^3^. For instance, according to the database PlantSSP^25^, there are 2451, 5373, and 3216 predicted SSPs, which are less than 200 aa in length with NSS, in *A. thaliana*, *O. sativa*, and *P. trichocarpa*, respectively. These predicted SSPs account for 6.9%, 8.0%, and 7.1% of all the annotated proteins (including splice variants) in the *A. thaliana* (version TAIR10), *O. sativa* (version MSU6.1), and *P. trichocarpa* (JGI v2) genome, respectively. More recently, with the release of the reannotated *M. truncatula* genome, 4439 genes (6.3% of all the annotated genes) were predicted to encode SSPs that are less than 230 aa with NSS but not transmembrane regions^3^. Although interest in decoding genomes for potential SSPs has been growing substantially in recent years, only a limited number of SSPs have been experimentally characterized, which are distributed among approximately 50 gene families^13^, with their representative members listed in Table 1.

**Table 1:** A list of representative small secreted proteins that have been experimentally confirmed in plants

Plant species	Protein name	Gene locus	Protein length (aa)	Gene family* (Pfam ID)	Reference
*Arabidopsis thaliana*	CEP1	AT1G47485	91		^89^
*Arabidopsis thaliana*	CLV3	AT2G27250	96	PF11250	^148^
*Arabidopsis thaliana*	EPLF9	AT4G12970	102	PF16851	^149^
*Arabidopsis thaliana*	EPF1	AT2G20875	104	PF13912	^150^
*Arabidopsis thaliana*	GLV6	AT2G03830	123		^151^
*Arabidopsis thaliana*	LTP1	AT2G38540	118	PF00234	^152^
*Arabidopsis thaliana*	PREPIP1	AT4G28460	72		^153^
*Arabidopsis thaliana*	PREPIP2	AT4G37290	84		^153^
*Arabidopsis thaliana*	PROPEP1	AT5G64900	92	PF00879	^154^
*Arabidopsis thaliana*	PROPEP2	AT5G64890	109	PF00879	^155^
*Arabidopsis thaliana*	PROPEP3	AT5G64905	96	PF00879	^55^
*Arabidopsis thaliana*	PSK1	AT1G13590	87	PF06404	^156^
*Arabidopsis thaliana*	RALF1	AT1G02900	120	PF05498	^157^
*Arabidopsis thaliana*	RGF1	AT5G60810	116		^158^
*Arabidopsis thaliana*	IDA1	AT3G25655	86		^159^
*Medicago truncatula*	NCR169	Medtr7g029760	61	PF07127	^160^
*Oryza sativa*	DEF7	LOC_Os02g41904.1	80	PF00304	^161^
*Populus trichocarpa*	CLE20	Potri.014G156600	74		^39^
*Solanum lycopersicum*	CAPE1	Solyc00g174340	159	PF00188	^162^
*Zea mays*	PROZIP1	AC210027.3_FG003	137		^51^

### Structure of known SSPs

**Fig. 3:**
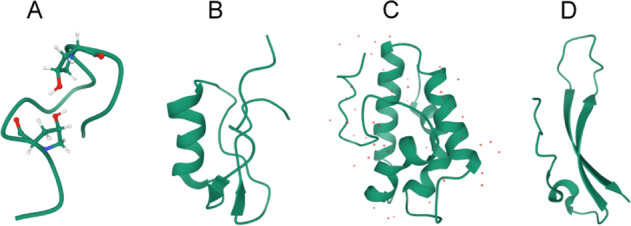
Three-dimensional structure of some known small secreted proteins in plants. **A** CEP1 (PDB ID: 2MFO). **B** SCR/SP11 (PDB ID: 1UGL). **C** LTP (PDB ID: 1MZL). **D** Stomagen (PDB ID: 2LIY). The Protein Data Bank (PDB) data were obtained from RCSB protein data bank (https://www.rcsb.org/)^165,166^ and visualized using Mol*^167^

**Fig. 4:**
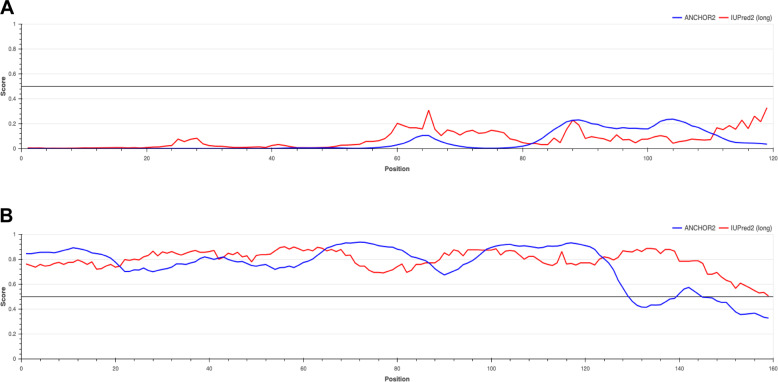
Examples of plant small secreted proteins containing intrinsically disordered regions (IDRs). **A** LTP1 (gene locus: AT2G38540), with a defined 3D structural domain (PDB ID: 1MZL). **B** LEA4 (gene locus: AT5G06760) with IDR only. The protein sequence data were obtained from Phytozome (https://phytozome-next.jgi.doe.gov/)^168^ and IDRs were predicted using IUPred2A (https://iupred2a.elte.hu/)^169,170^

Protein function is dependent on a well-defined and folded three-dimensional (3D) structure and intrinsically disordered regions (IDRs), which are not likely to form a defined 3D structure^26^. Some of the known SSPs in plants have well-defined 3D structure, as demonstrated in Fig. 3. For instance, hydroxyproline-bound tri-arabinoside-induced conformation was found when post-translationally modified protein CLV3 became biologically active^27^. The β-turn-like conformation, for example, which is a feature of CEP1, is associated with biological activity^28^. On the other hand, enzymatic maturation processes produce bioactive Cys-rich SSPs with correct oxidative folding under oxidative conditions by

forming diverse disulfide patterns as well as loop regions, which are supposed to be crucial for protein–protein interactions (PPIs)^15,29^. SCR/SP11 contains an α/β sandwich motif connected by L1 loop that serve as binding site for specific receptors^30^. LTP has four α-helices, three loops, and four disulfide bridges with eight conserved cysteines^4^. EPF includes one loop and three disulfide bonds, which contains two antiparallel β-strands connected by a 14-residue loop^31^. However, it has been estimated that 10% of secreted proteins are intrinsically disordered proteins (IDPs), with >70% of their length being IDRs^26^. For example, LTP1 from *A. thaliana* contains a defined 3D structural domain (Fig. 3C) and without IDR (Fig. 4A) but LEA4 from *A. thaliana* has no defined 3D structural domain and is fully disordered (Fig. 4B).

### Biological roles of known plant SSPs

#### Role of SSPs in plant growth and development

Some of the known SSPs are associated with multiple aspects of plant growth and development. During these processes, most SSPs act as signaling molecules that are involved in cell-to-cell communication by binding membrane receptors and coordinating responses with plant hormones^14,32^. In terms of meristem maintenance, CLE14 and CLE40 expression has been observed in *A. thaliana* root meristematic zone and found to play roles in controlling meristematic activity as well as cell number^33,34^. Although CLE43 does not affect root apical meristem growth in *A. thaliana*^35^, its homologs, BnCLE43a and BnCLE43b, were found in *Brassica napus* could repress *A. thaliana* root growth when synthetic peptides were added to the culture medium^36^. In *A. thaliana*, both CLE9 and CLE10 control xylem differentiation through regulation of the cytokinin signal pathway^37^, and CLE41 can drive vascular cell division^38^. In contrast, PtrCLE20 identified in vascular cambium cells of *P. trichocarpa* was shown to restrain cell division, resulting in an inhibition of lateral growth of the stem^39^. Besides the impact on vegetative tissues or organs, SSPs can affect flower development. For example, CLV1 acts with CLV3 to avoid enlarged meristems and extra floral organs in *A. thaliana*^40^. The pollen-specific *SlPRALF* gene that encodes a 129 aa preproprotein was recognized to negatively regulate pollen tube elongation in *S. lycopersicum*^41^.

#### Role of SSPs in plant response to abiotic and biotic stresses

To sense and respond to various stresses, plants have evolved complex signaling and defense mechanisms^42^. Induced SSPs have been observed in many stress responses in plants, including some SSPs recognized as hormone-like molecules^43^. SSPs act quickly and synergistically at low concentrations in reaction to different stresses^44^.

SSPs are involved in a variety of biotic stresses responses in diverse plant species. For example, an SSP called SYSTEMIN identified in *S. lycopersicum* was the first wound response signaling peptide^45,46^. When plants are attacked by herbivores or pathogens, a series of defense signals and pathways can be induced by SYSTEMIN through its interaction with SYSTEMIN RECEPTOR 1, which includes stimulation of PROTEASE INHIBITOR production, as well as enhancement of ethylene and jasmonic acid biosynthesis^47,48^.

Plant SSPs can initiate immune responses and increase resistance to pathogens. For example, an SSP called IRP, which was identified from the proteomic analysis of *O. sativa* suspension cells cultured with bacterial peptidoglycan and fungal chitin, increased the abundance of phenylalanine ammonia-lyase 1 (PAL1) and activated mitogen-activated protein kinases (MAPKs), which are known to be associated with plant immunity^49^. Two pathogen-responsive SSPs, TaSSP6 and TaSSP7, are responsible for resistance to Septoria tritici blotch, a severe foliar disease caused by the fungal pathogen *Zymoseptoria tritici* in *Triticum aestivum*^50^. In *Z. mays*, Zip1 was demonstrated to trigger plant immunity by activating salicylic acid defense signaling^51^.

SSPs are also involved in responses to abiotic stresses. For example, CLE25, found in *A. thaliana*, is induced under dehydration, which triggers ABA biosynthesis in leaves to prevent water loss by regulating stomatal closure^52^. In *A. thaliana* roots, *AtRALFL8* encoding a SSP can be induced not only by nematode infection but also by drought stress, leading to cell wall remodeling^53^. To determine extracellular proteins that respond to heat stress, a quantitative proteomic analysis was conducted by collecting proteins from heat-tolerant *Sorghum bicolor* cell suspension culture medium, resulting in the identification of an SSP named germin protein, which was highly induced at the protein level^54^. Another example is the small peptide AtPep3 encoded by *AtPROPEP3*, which has been shown to play an important role in salinity stress tolerance in *A. thaliana*^55^.

#### Role of plant SSPs in beneficial plant–microbe interactions

SSPs play important roles in cross-kingdom interactions. It is widely accepted that SSPs generated from plant-associated microorganisms (e.g., fungi, bacteria) can be used as effector proteins to promote plant microbial colonization^56–58^. However, studies on the identification of plant SSPs as effector proteins that affect microbes have been very limited^2^. Plants can adapt to a low availability of nutrients by altering root system architecture, with some can form symbiotic associations with rhizobia and mycorrhizal fungi^59,60^. In legumes, SSPs can affect root development and rhizobial–legume symbiosis^61,62^. CLE family members have been characterized in different species, such as CLE12 and CLE13 in *M. truncatula*, CLE-RS (CLE-root signal) 1/2/3 in *Lotus japonicus,* and RIC (rhizobium-induced CLE) in *G. max*. These SSPs appear to be involved in the negative systemic autoregulation of the nodulation pathway and inhibit newly formed nodules in roots^63^. Conversely, in *M. truncatula*, CEP1 was found to modulate lateral root formation and increase the number and size of nodules^60^. When *L. japonicus* was inoculated with the arbuscular mycorrhizal (AM) fungus *Rhizophagus irregularis*, in comparison with formation of nodules in *L. japonicus*, alternate CLE genes, including *LjCLE19* and *LjCLE20*, were upregulated in roots, indicating that different signaling pathways are involved in AM and root nodule symbiosis^64^. In addition, a recent study reported that SSPs produced by *P. trichocarpa* were induced when co-culture with ectomycorrhizal mycorrhizal (EM) fungus *Laccaria bicolor* and several *P. trichocarpa* SSPs could enter fungal hyphae when they were exposed to *L. bicolor*^2^, suggesting plant SSPs may mediate ectomycorrhizal symbiosis as well.

## Computational and experimental approaches for discovery of SSPs in plants

### Computational approaches for discovery of SSPs

In general, there are two main steps to computationally predict SSPs in plant genomes, i.e., predicting small proteins encoded by sORFs and subsequently evaluating their ability to be secreted. A large number of sORFs can be found by locating in-frame start and stop codons in the plant genomes. However, annotations of sORFs have been largely overlooked because such short sequences were initially classified as random nonsense occurrences^65^. In the recent decade, progress in the development of computational methods for gene prediction has contributed to the identification of numerous sORFs in plants. For example, sORF finder is a tool for identifying putative small sORFs between 10 and 100 amino acids based on significant selective constraints, which works well for predicting sORFs in plant genomes^66^. Small Peptide Alignment Discovery Application is a homology-based program which can accurately identify and annotate genes in a given family, including sORFs in plants^67^. One caveat of these in silico sORF prediction tools is that the predicted sORFs may be pseudogenes. To address this issue, transcript expression data generated by transcriptome sequencing (RNA-seq) can be used for identifying functional sORFs, as demonstrated in SSP discovery in *P. trichocarpa*^2,10^. Transcript sequences obtained from RNA-seq data can be either protein coding sequences (CDS) or non-coding RNAs^68,69^. Finally, using DeepCPP, a new deep neural network-based tool, aims to predict short sequences with coding potential^70^.

The potential for secretion of small proteins has been determined using tools based on specific algorithms, in particular many use newly developed machine learning (ML) approaches (Table 2). To predict NSS-containing SSPs, SignalP 5.0, based on deep neural networks, is commonly utilized because it has a user-friendly interface and good performance across plant species^71^. However, since an NSS is common in several types of membrane proteins, membrane spanning proteins with both predicted signal peptide and at least one transmembrane region should be excluded^72^. MEMSAT-SVM^73^ can be used for transmembrane helix topology prediction, and SPOCTOPUS^74^ is designed for predicting both signal peptide and transmembrane topology. Because the existence of certain numbers of NSS-containing proteins follow UPS routes, SecretomeP has been constructed and is a ML algorithm to predict unconventionally secreted proteins^75^. In addition, the number of Cys residues and their arrangement have been used to predict Cys-rich SSPs without signal peptide^76^. In some studies, an additional criterion, such as the lack of endoplasmic reticulum-retention motif, is taken into consideration for secretion prediction. Several authors recommend that small proteins containing C-terminal KDEL or HDEL motifs should be excluded as non-SSPs^76,77^. Protein secretion mediated by conventional (e.g., CLE^78^) or unconventional (e.g., PME^79^) mechanisms can be evaluated using various tools for predicting multiple protein subcellular localizations, such as LocTree3 (refs. ^80,81^), CELLO^82^, YLoc^83^, DeepLoc^84^, and TargetP^85^. Also, ML-based methods have been developed recently for predicting both conventional and unconventional secretion, e.g., ApoplastP^86^, BUSCA^87^, and Plant-mSubP^88^. A pipeline integrating the best methods for computational prediction of SSPs is proposed in “Integrative approaches for discovery of SSPs”.

**Table 2:** A list of representative computational resources and tools for predicting plant small secreted proteins

Type	Name	Description	Website	Reference
Database	MtSSPdb	SSPs in *Medicago truncatula*	https://mtsspdb.noble.org	^3^
Database	PlantSecKB	All secreted proteins in multiple species	http://proteomics.ysu.edu/secretomes/plant.php	^163^
Database	OrysPSSP	SSPs in *Oryza sativa*	http://www.genoportal.org/PSSP/index.do	^5^
Standalone Package	DeepCPP	Predicting RNA coding potential	https://github.com/yuuuuzhang/DeepCPP	^70^
Online tool	SignalP 5.0	Predicting signal peptides	http://www.cbs.dtu.dk/services/SignalP/	^71^
Online tool	SecretomeP	Predicting non-classical protein secretion	http://www.cbs.dtu.dk/services/SecretomeP/	^75^
Online tool	OutCyte	Predicting unconventional protein secretion	http://www.outcyte.com/	^164^
Online tool	ApoplastP	Predicting effectors and plant proteins in the apoplast using machine learning	http://apoplastp.csiro.au	^86^
Online tool	DeepLoc	Prediction of subcellular localization of eukaryotic proteins	http://www.cbs.dtu.dk/services/DeepLoc/	^84^
Online tool	LocTree3	Predicting subcellular localizations	https://rostlab.org/services/loctree3/	^80^
Online tool	BUSCA	Predicting subcellular localizations	http://busca.biocomp.unibo.it	^87^
Online tool	Plant-mSubP	Predicting subcellular localizations	http://bioinfo.usu.edu/Plant-mSubP/	^88^
Standalone package	MEMSAT-SVM	Transmembrane helix topology prediction; identifying the cytosolic and extracellular loops.	https://github.com/psipred/MemSatSVM	^73^

### Experimental approaches for discovery of SSPs

The putative SSPs predicted using computational approaches described in “Computational approaches for discovery of SSPs” need to be verified using experimental approaches to provide protein-level evidence. To address this issue, protein mass spectrometry (MS) data can be used to determine (1) whether the predicted SSPs are truly expressed proteins in extracellular localization and (2) whether the predicted SSP sequences are full length or partial fragments of longer protein sequences. For instance, a novel 15 aa secreted peptide named CEP1 encoded by AT1G47485 was effectively identified in *A. thaliana* by liquid chromatography-mass spectrometry (LC-MS) analysis^89^. The feasibility of this system was tested initially by detecting a known small secreted peptide CLE44 in the medium using transgenic *A. thaliana* overexpressing the CLE44 gene. Computational prediction of SSP secretion can also be verified through MS analysis of extracellular proteins. For example, protein MS has been successfully used to identify plant immune response proteins that are secreted into apoplastic space in *A. thaliana* leaves^90^. Proteomic analyses of secretomes have identified secreted proteins in *O. sativa*^91^, *Hippophae rhamnoides*^92^, *S. bicolor*^54^, *Solanum chacoense*^93^, and *S. lycopersicum*^94^. Such global analyses of plant secretomes could facilitate the discovery of SSPs. However, proteins containing IDRs of sufficient length tend to be more susceptible to degradation, resulting in lower protein abundance^26^. This may cause a problem for studying plant SSPs that contain a large portion of IDRs using proteomics approaches because MS has lower sensitivity than transcriptome sequencing. To increase the sensitivity of detecting SSPs in plants, it is necessary to enrich for IDRs containing proteins and low molecular weight proteins in protein extract using gel filters^95^ or ultrafiltration devices^96,97^.

Besides plant secretome proteomics, molecular approaches can be used to test SSP secretion. For example, the CDS of SSPs can be fused with reporter genes, such as green fluorescent protein^98^, and the gene fusion constructs can be tested for secretion of reporter-tagged SSPs using agroinfiltration-based transient gene expression^99^ or stable transformation in plants. The secretion of SSPs has been tested using the yeast expression system as well^2^.

### Integrative approaches for discovery of SSPs

**Fig. 5:**
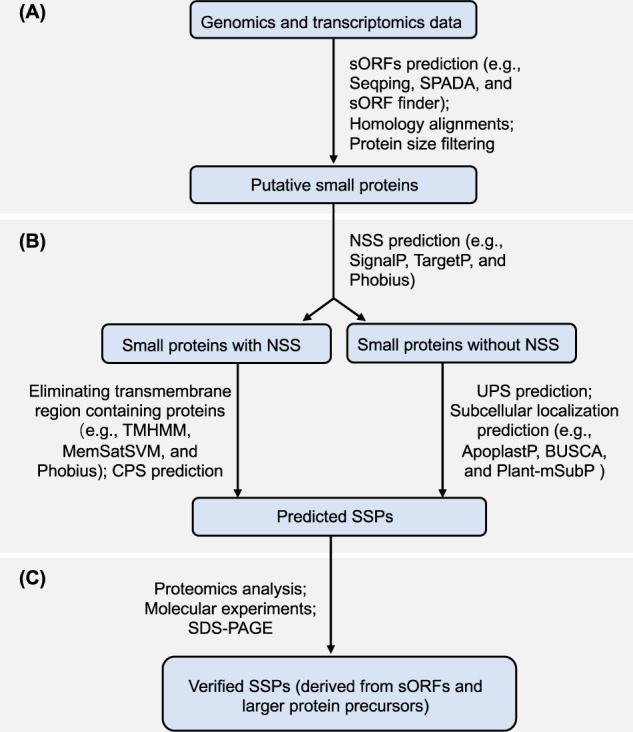
**A**n integrative pipeline for discovery of small secreted proteins (SSPs) in plants. **A** Small open reading frames (sORFs) encoding small proteins can be predicted by using gene prediction tools based on genome sequence and transcriptomic data. **B** Predicting secretion processes for small proteins using machine learning approaches. **C** Experimental validation of predicted SSPs. NSS: N-terminal signal sequence for protein secretion, CPS: conventional protein secretion, UPS: unconventional protein secretion, MS: mass spectrometry, SDS-PAGE: sodium dodecyl sulfate polyacrylamide gel electrophoresis

From an amalgamation perspective, multiple tools can be assimilated to predict SSPs. Here we propose such a pipeline for SSP discovery by integrating the methods discussed in Sections “Computational approaches for discovery of SSPs” and “Experimental approaches for discovery of SSPs” (as illustrated in Fig. 5). Briefly, sORFs encoding small proteins are predicted from genomic sequences using gene prediction pipeline such as Seqping^100^ based on self-training HMM models and transcriptomic data. Next, NSS-containing small proteins that are transported via CSP pathways are predicted with ML-based tools, such as SignalP 5.0. At this stage small proteins containing transmembrane regions, which are unlikely to be secreted, should be identified and eliminated from downstream analysis. Given that some NSS-containing proteins follow USP pathways, additional ML-based software, such as SecretomeP, may be applied simultaneously. In addition, the secretion ability of proteins without an NSS are inferred by subcellular localization prediction tools (Table 2), which are helpful for predicting secreted proteins contaning an NSS as well. Putative SSPs predicted by computational tools are then validated with MS-based and/or molecular experiments, particularly for their secretion ability, before further functional characterization. Proteomics data are then used to confirm the protein expression of putative sORFs to discover small proteins that are derived from larger protein precursors and/or to localize protein accumulation outside cells.

## Strategies for elucidating the function of plant SSPs

### Examination of secretion and transport pathways

Given that apoplastic localization of SSPs can be vital for their function, functional characterization of SSPs often requires refining the knowledge of their trafficking, transport, and secretion routes both within plants and between plants and their microbial partners. Perhaps the most direct method for investigating SSP movement is to visualize SSPs under a fluorescence or electron microscope after tagging them with a fluorescent protein or other label, as demonstrated by Wang et al.^101^ when investigating EXPO-mediated transportation of the *A. thaliana* Exo70 paralog—Exo70E2, and by Chen et al.^102^ when studying the movement of the transcription factor HY5 from shoot to root in *A. thaliana*. One requirement for this approach is that the fusion of the SSPs and the fluorescent markers must not alter the mobility, secretion, or the function of the SSPs^23,103^ or interfere with the folding and fluorescence intensity of the markers.

Small-molecule reagents have been used to dissect protein trafficking routes. A widely used example is the fungal toxin brefeldin A (BFA). Given that BFA can disrupt the retrograde traffic from the Golgi to the ER, it serves as a powerful tool for distinguishing Golgi-dependent and -independent protein trafficking^104,105^. Another example is concanamycin A (ConcA)—an inhibitor of vacuolar-type ATPase (V-ATPase), which blocks post-Golgi trafficking and has been used in examining the transportation pathway of VHA-a3 (refs. ^106,107^). Additionally, small molecules that can interact with trafficking-related organelles or vesicles have been used to screen for their potential application in elucidating protein secretion pathways^108^. The power of these trafficking inhibitors, however, becomes limited when it comes to examining the movement of SSPs between plants and microbes. An alternative approach could be based on fluorescently tagged SSP, which was discussed above and appears to be more useful for examining the cross-kingdom movement of plant SSPs.

In addition, a learn-by-design approach based on rewriting the transport pathway can be informative for evaluating if secretion is required for SSP function. Targeted redirection has been achieved by fusing SSPs to alternative sorting signals. For example, Rojo et al.^109^ fused different vacuolar sorting signals to the C terminus of CLV3 and redirected the destination of CLV3 from apoplast to the vacuole. The authors concluded that apoplastic localization is essential for CLV3 to activate the CLV signaling pathway in *A. thaliana*.

### Uncovering phenotypic traits conferred by SSP-encoding genes

Reverse genetics techniques, by imparting loss- or gain-of-function mutations via ectopic expression, virus-induced gene silencing, and RNA interference (RNAi)^110,111^, are among the most powerful tools to reveal phenotypes associated with genes of interest. These techniques work equally well for studying the function of SSP-encoding genes. For example, CLV3—the meristem development regulator, when constitutively overexpressed in transgenic *A. thaliana*^112^ demonstrated the correlation between the level of CLV3 protein and the accumulation of the meristem cells. In addition, *A. thaliana* in which the expression of CLV3 was suppressed by RNAi was created by Chuang and Meyerowitz^113^ for studying the associated phenotypic changes in floral development. Similarly, RNAi-induced suppression of the *PtCLV3* ortholog *PttCLE47* were employed by Kucukoglu et al.^114^ to investigate its role in cambial development and secondary xylem formation in hybrid aspen (*Populus tremula* *×* *P. tremuloides*).

Besides traditional techniques, the recent revolution in gene editing tools, particularly the invention of the CRISPR/Cas and related technologies, provides new opportunities for efficient gene knockout, gene knockin, gene activation, and gene suppression in plants^115–118^. Its development is based on an immune system naturally found in bacteria and archaea, the CRISPR/Cas9 system has been widely used for creating gene knockouts by creating double-strand breaks, which are then repaired by error-prone the non-homologous end joining in plants and therefore often lead to indel mutations in the target gene. The efficacy of CRISPR/Cas9-mediated gene knockout has been demonstrated in a number of herbaceous and woody plant species^119–122^. In the last few years, the adaptation of CRISPR into a recruiting platform and the discover of Cas9 variants have made CRISPR/Cas a more versatile tool. For example, transcriptional activation and suppression of single and multiple genes can now be conferred by the CRISPR/deactivated Cas9 (dCas9)-based transcriptional regulation system^123,124^. All of these tools can be used in tuning the expression of SSPs for revealing their targets and examining their biological impacts.

### Identification of receptors and partners involved in SSP signal transduction pathways

As discussed above (see “Biological roles of known plant SSPs”), many plant SSPs act as signaling molecules and have the ability to affect the expression of other genes. Therefore, identifying the receptors and other downstream targets of an SSP of interest is the ultimate step towards deciphering SSPs’ biological function. A number of early studies, particularly those done in *A. thaliana*, have been relying on creating targeted mutants or performing mutational screen to achieve this goal. Taking receptors of CLV3 in *A. thaliana* for instance: CLV1, which is a leucine-rich repeat receptor-like kinase, was verified via phenotypic analysis of single or double mutants^125^. Meanwhile, CORYNE (CRN) which is a membrane-associated protein kinase, and TOADSTOOL2 (TOAD2) which is a receptor-like kinase, were identified by screening the population created with ethyl methanesulfonate mutagenesis^126,127^.

Besides mutational screens, PPI data can provide valuable evidence in identifying novel partners that interact with SSPs during signal transduction. Several in vitro and in vivo PPI detection approaches, such as affinity purification (AP), tandem affinity purification, and yeast two-hybrid (Y2H), have been commonly used^128^. In particular, the capability of Y2H-based approaches has been extended from one-by-one clonal identification to proteome-wide mapping of PPIs, with the recent development of matrix-based Y2H methods coupled with next-generation sequencing (NGS) technology^129^. Compared with mutational screen, Y2H-NGS approaches make it possible to identify novel interaction partners of SSPs even within an organism whose genome has not been fully annotated yet.

### Discovery-based extraction, screening, and identification of SSPs

**Fig. 6:**
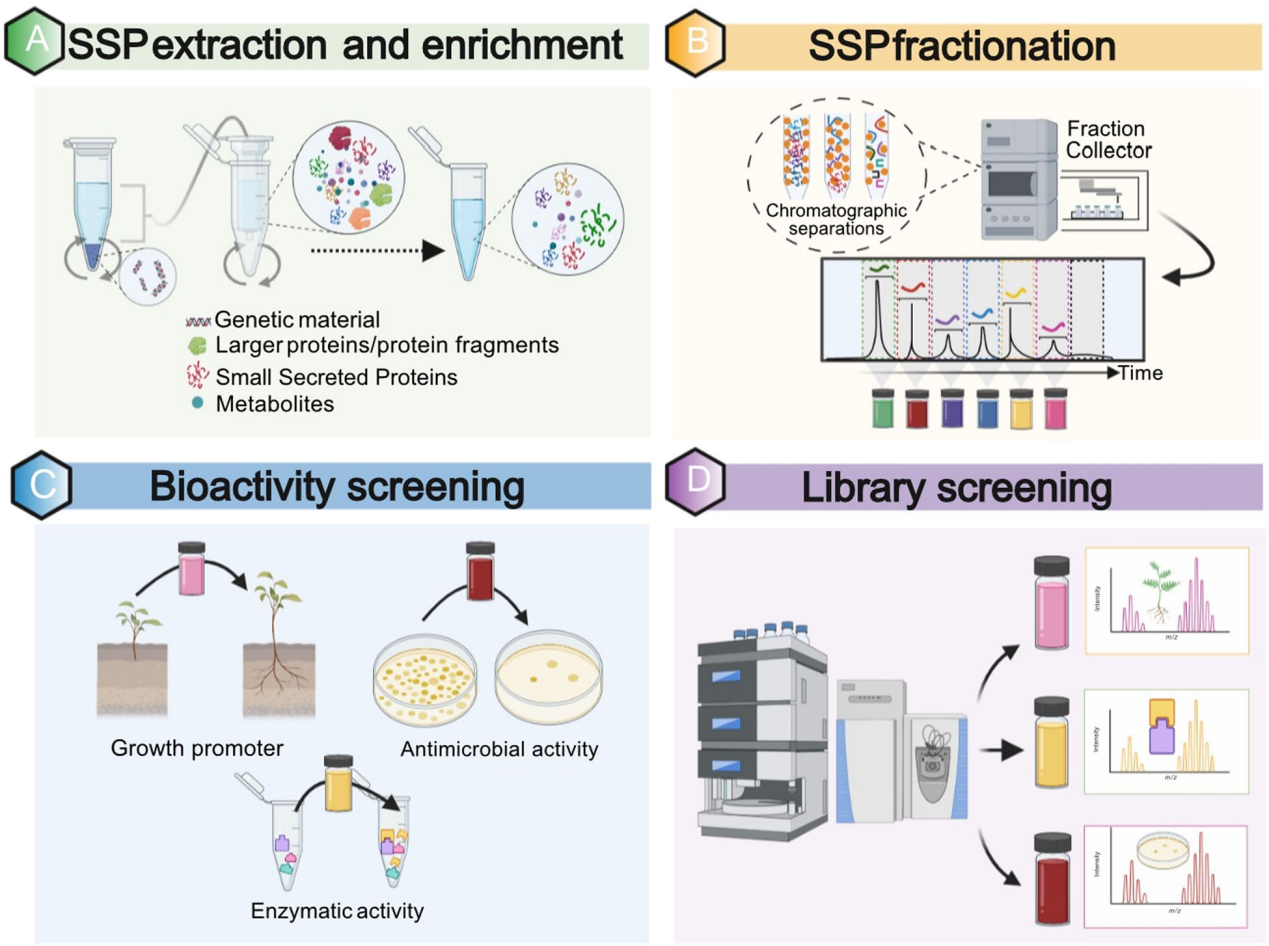
Experimental framework to screen biologically relevant small secreted proteins (SSPs). The experimental workflow to characterize bioactive SSPs consists of four main steps: **A** The extraction and enrichment of the low molecular weight (MW) fraction of the secreted proteome of a sample, e.g., with the use of molecular weight cut-off filters. **B** The fractionation/isolation of low MW fractions using different chromatographic separations techniques to reduce their complexity and assemble a set of SSP candidates to test for bioactivity. Other low MW molecules like metabolites can be removed at this step if needed. **C** SSP bioactivity assays against cell-based or cell-free systems to elucidate their mechanisms of action (i.e., growth promotion or antimicrobial activity). **D** Interrogation of SSP fraction libraries with bioactivity via high-resolution/high-mass accuracy LC-MS/MS. Novel SSP sequence characterization could be aided by *de novo* search strategies. Figure was created with BioRender.com

High-throughput analytical approaches that couple selective enrichment, fractionation/isolation, and phenotype screening followed by MS-based identification provide an established framework to screen plant tissues for biologically relevant SSPs^45,89,130–132^ (Fig. 6). This classical approach for the discovery of novel natural products starts with an enrichment strategy to selectively isolate molecules of interest from highly complex crude extracts. For SSPs, common cellular extraction techniques use size exclusion ultrafiltration strategies, such as molecular weight cut-off spin column filters, to selectively enrich for low molecular weight protein fractions^96,97^. Other techniques include gel-based separations^49,95,133^, solvent extractions^89,134^, and size exclusion chromatography^134,135^. Following these enrichment strategies, SSPs can be further fractionated based on physicochemical properties (e.g., polarity, hydrophobicity, stability, solubility) using liquid chromatography^136–138^.

Either as crude extract mixtures, enrichments, or isolated fractions, SSPs can be evaluated for their bioactivity against cell-based or cell-free biosystems. Cell-based screening can be used to assess simple effects on cell viability, morphology, and proliferation, or to elucidate the mechanism of action. Common phenotypes profiled in cell-based systems are growth promotion/restriction or antimicrobial activity^139–142^. Alternatively, cell-free screening has been employed to evaluate the effect of SSPs to better describe the thermodynamic, kinetic, or structural basis for molecular interactions with other cellular constituents^143^. Cell-free screening can be employed to identify SSPs with the abilities to scavenge free radicals, chelate metals, or bind to certain macromolecular targets that regulate various biological processes such as epigenetic processes and cell proliferation^144,145^.

Following the detection of fractions with relevant bioactivity, molecule libraries can be further interrogated via high-throughput LC-MS/MS to sequence unknown SSPs. Some of the current challenges in accurate and sensitive identification of SSPs with MS include lack of SSP representation in protein databases, inadequate understanding of SSP maturation mechanisms, and partial knowledge of their PTM. Thus, the characterization of SSPs by LC-MS/MS can benefit from the use of *de novo* search strategies^146^. *De novo* sequencing algorithms derive peptide sequences using only fragment ion information from the tandem mass spectra, are generally optimized to run without the restriction of cleavage enzymes (i.e., trypsin) and work in an unbiased manner as they do not necessarily require any input based on prior knowledge of the sample^147^.

## Conclusion and perspectives

In the past several years, there has been increasing evidence that SSPs play important roles during plant growth, development and response to biotic and abiotic stresses, and consequently a growing appreciation of the biological significance of plant SSPs. A sheer number of SSPs have been predicted in diverse lineages of organisms, and the intercellular or inter-organismal movement of SSPs infers that SSPs are likely a significant and common mode of signaling among organisms. It is now known that SSPs are synthesized and secreted via diverse pathways in plants. Currently, however, the number of characterized SSPs in plants is low. The majority of SSPs encoded in plant genomes are overlooked and remain unannotated. Roadblocks that prevent progress in the study of SSPs include (1) a lack of reliable methods for isolating SSPs for experimental characterization, (2) a lack of capabilities for real-time monitoring the intercellular or inter-organismal movement of SSPs, (3) a lack of structural data for SSPs, and (4) a lack of computational tools for predicting non-conventional secretion of SSPs.

Recent advances in high-throughput molecular screening approaches and bioinformatics offer exciting opportunities for the discovery and characterization of SSPs. For example, the rapid accumulation of omics data, including genomics, transcriptomics, and proteomics, provide rich databases for discovering plant SSPs, including those derived from larger protein precursors and directly encoded by sORFs. Meanwhile, advanced ML tools have evolved to predict the secretion pathways, including both CPS and UPS that SSPs follow. Such computational prediction on secretion can be verified experimentally, for example, via bioimaging of fluorescent reporter-tagged protein candidates. In addition, advanced plant biotechnologies, particularly, CRISPR/Cas-based genome-editing systems and transcriptional regulation systems (i.e., CRISPRa and CRISPRi) allow for efficient gene knockout, activation, and suppression, and therefore analysis of the biological roles of SSPs, and identification of their partners by combining with PPI and NGS data. The discovery and functional role of SSPs in plant growth and development will continue to expand in the near future.
